# Supplementary material for: Detailed molecular and epigenetic characterization of the pig IPEC-J2 and chicken SL-29 cell lines
Source: iScience. 2023 Feb 20;26(3):106252. doi: 10.1016/j.isci.2023.106252 (PMC10018572; doi:10.1016/j.isci.2023.106252)
Supplement: Data S2. Complete homer output for identified motifs in Chicken SL-29, related to Tables 5 and 6 — Homer motif analysis results for histone modifications H3K4me1, H3K4me3, H3K27ac, enhancers, and ATAC-seq of chicken SL-29 cell line. Parameters for possible false positives is as mentioned earlier for S5. [file mmc3.zip › Data_S2/S6/Chicken_SL_29/peak_files_H3K4me3/homerResults/motif1.similar.html]

motif1

## Information for motif1

T
C
A
G
C
T
G
A
C
G
T
A
C
G
T
A
T
C
A
G
G
C
A
T
C
T
A
G
C
G
T
A
C
G
T
A
C
T
G
A
T
A
C
G
G
A
C
T
C
T
G
A
  
Reverse Opposite:  

G
A
C
T
C
T
G
A
A
T
G
C
A
G
C
T
A
C
G
T
A
C
G
T
A
G
T
C
C
G
T
A
A
G
T
C
A
C
G
T
A
C
G
T
A
G
C
T
A
G
T
C
  

|  |  |
| --- | --- |
| p-value: | 1e-232 |
| log p-value: | -5.355e+02 |
| Information Content per bp: | 1.727 |
| Number of Target Sequences with motif | 583.0 |
| Percentage of Target Sequences with motif | 3.40% |
| Number of Background Sequences with motif | 198.0 |
| Percentage of Background Sequences with motif | 0.61% |
| Average Position of motif in Targets | 148.8 +/- 81.4bp |
| Average Position of motif in Background | 161.5 +/- 99.9bp |
| Strand Bias (log2 ratio + to - strand density) | 0.1 |
| Multiplicity (# of sites on avg that occur together) | 1.06 |
| Motif File: | file (matrix) reverse opposite |

### Similar de novo motifs found

|  |  |  |  |  |  |  |  |
| --- | --- | --- | --- | --- | --- | --- | --- |
| Rank | Match Score | Redundant Motif | P-value | log P-value | % of Targets | % of Background | Motif file |
| 1 | 0.912 | T A G C A C G T G A C T A C G T G T A C C T A G A G T C A C G T G A C T G A C T | 1e-225 | -518.239520 | 5.53% | 1.60% | motif file (matrix) |
| 2 | 0.899 | C G T A C G T A T C A G A C G T A C T G C G T A C G T A C G T A | 1e-82 | -190.778308 | 8.94% | 5.32% | motif file (matrix) |
| 3 | 0.656 | A T G C A C T G A G C T A C G T A G T C A T C G A C G T A C G T A G C T A G C T | 1e-55 | -128.092659 | 17.86% | 13.57% | motif file (matrix) |
| 4 | 0.754 | A C G T A C G T A C T G A G T C A C G T A C G T A C G T A G T C A C T G A G T C A C G T A C G T A C G T | 1e-54 | -125.769586 | 13.26% | 9.57% | motif file (matrix) |
| 5 | 0.706 | A C T G A G C T A C G T A C G T A G T C G C T A A G T C G A C T A C G T A G T C C A T G A T C G A G C T | 1e-17 | -39.367832 | 0.11% | 0.01% | motif file (matrix) |
